# Effects of chronic volume deprivation on the ventricle

**DOI:** 10.1093/icvts/ivaf124

**Published:** 2025-06-03

**Authors:** Bjorn Cools, Filip Rega, Alexander Van De Bruaene, Manon Van Hecke, Libera Fresiello, Stephanie Devleeschauwer, Stephen Brown, Piet Claus, Marc Gewillig

**Affiliations:** Department of Pediatric and Congenital Cardiology, University Hospitals Leuven, Leuven, Belgium; Department of Cardiovascular Sciences, Catholic University of Leuven, Leuven, Belgium; Department of Cardiovascular Sciences, Catholic University of Leuven, Leuven, Belgium; Department of Cardiac Surgery, University Hospitals Leuven, Leuven, Belgium; Department of Cardiovascular Sciences, Catholic University of Leuven, Leuven, Belgium; Department of Adult Congenital Cardiology, University Hospitals Leuven, Leuven, Belgium; Department of Imaging & Pathology, Catholic University Leuven, Leuven, Belgium; Department of Cardiovascular Sciences, Catholic University of Leuven, Leuven, Belgium; Department of Biomedical Imaging & Sensing, Faculty of Science and Technology, University of Twente, Twente, The Netherlands; Animal Research Center, Catholic University Leuven, Leuven, Belgium; Department of Pediatric and Congenital Cardiology, University Hospitals Leuven, Leuven, Belgium; Department of Pediatric and Congenital Cardiology, University of the Free State, Bloemfontein, South Africa; Department of Cardiovascular Sciences, Catholic University of Leuven, Leuven, Belgium; Department of Pediatric and Congenital Cardiology, University Hospitals Leuven, Leuven, Belgium; Department of Cardiovascular Sciences, Catholic University of Leuven, Leuven, Belgium

**Keywords:** preload, ventricular volume deprivation, muscular stretch, volume reloading, animal model

## Abstract

**OBJECTIVES:**

Little is known of the haemodynamic changes following chronic ventricular volume deprivation, which impact understanding the disease course and treatment results. An animal model was created to study the effects of chronic ventricular volume deprivation and acute reloading.

**METHODS:**

In 13 lambs, a polytetrafluoroethylene strip was placed around the inferior and superior caval vein through thoracotomy resulting in progressive ventricular volume deprivation during growth. After 10 months, the polytetrafluoroethylene bands were relieved. Magnetic resonance imaging and haemodynamic measurements including pressure–volume loops were performed before and after debanding and compared to age and weight-matched controls (*n* = 6).

**RESULTS:**

The end-diastolic pressure was elevated compared to healthy animals (median [interquartile range] 8.1 [7.2–9.1] vs 1.0 [1.0–2.7] mmHg, *P* 0.030). The end-diastolic pressure after debanding increased to 11.1 (10.4–17.2) mmHg, *P* 0.038. The end-diastolic volume and end-systolic volume of the intervention group were also significantly less than the healthy controls (71.5 [66.7–74.7] vs 81.5 [74.3–86.3] ml, *P* 0.004 and 34.5 [27.5–37.6] vs 42.7 [35.0–50.5] ml *P* 0.001). The end-diastolic pressure–volume relationship was significantly shifted upwards and to the left compared to controls, indicative of a decreased compliance of the chronically deprived left ventricle. Histologic assessment revealed no significant differences in fibrosis between the ventricles of the intervention group and healthy animals.

**CONCLUSIONS:**

When a healthy ventricle is chronically deprived of an adequate preload, it becomes less compliant with elevated filling pressures. Acute reloading does not lead to ventricular systolic dysfunction, but in the early phase, diastolic pressure may rise. A better understanding of this phenomenon might help to recognition and treatment of impaired ventricular compliance.

## Graphical Abstract

**Figure:**
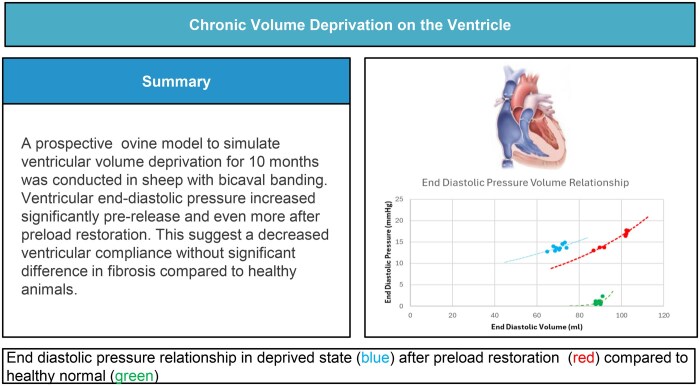

## INTRODUCTION

Extensive research has been conducted to evaluate the adaption of the ventricle when it becomes overstretched and dilated due to chronic volume or pressure overload, both clinical conditions commonly encountered in cardiologic practice. At the other end of the loading spectrum, the effects of chronic volume deprivation of the systemic ventricle have barely been studied [1, 2].

Reduced preload can occur in an acute state such as in severe blood loss (e.g. trauma) and dehydration [3, 4]. However, little is known regarding cardiovascular adaptations resulting from a chronically reduced preload. Chronic volume deprivation can be observed in several clinical conditions such as constrictive pericarditis, severe right heart dysfunction, Mustard baffle obstruction, the Fontan operation, large atrial septal defects and mitral stenosis [5, 6]. The causes of chronic preload deprivation are multiple, and it may be located at the level of the left atrioventricular valve, the atria through a left-to-right shunt or restrictive baffle, the pulmonary circulation because of elevated pulmonary vascular resistance or a poor-functioning or absent subpulmonary ventricle. These will act as bottlenecks in the circulation upstream of the left ventricle (LV), resulting in chronic preload starvation of that downstream ventricle. Secondary changes of the ventricle may influence the disease and/or treatment.

In striated muscles limiting the range of motion by splinting or disuse leads to increased muscle stiffness, most likely due to reduced or absent stretching from the new baseline. It has been documented that passive stretch of normal muscle produces a transient reduction in stiffness (viscoelastic stress relaxation) that persists for 1 to 2 hours before returning to pre-stretch levels [7–9]. Stiffness is a continuously adapting property of skeletal muscles, adjusting daily to the experienced range of motion [7]. Consequently, one can predict that chronic volume deprivation will result in decreased ventricular compliance and increased filling pressures. This prediction can be inferred from several clinical observations but has never been studied as such.

The study aimed to create a chronic volume-deprived ventricle in an animal model and study the effects of chronic volume deprivation and acute reloading.

## MATERIALS AND METHODS

The study was conducted in the animal laboratory facility of the Department of Experimental Cardiac Surgery (licence number LA1210253) using a large animal ovine model and approved by the ethical review board (P032-2010). All experiments were conducted in accordance with the legislation on animal welfare as indicated in Appendix XI of the Royal Decree of 29 May 2013 concerning the protection of laboratory animals.

Thirteen sheep were included in the intervention group. Two animals demised after the banding procedure: one with massive ascites most likely to a too tight inferior vena cava (IVC) banding and another during the debanding procedure due to rupture of the IVC. The remaining 11 animals and a group of 6 age and weight-matched animals were used for the analysis. All were analysed by means of magnetic resonance imaging (MRI) scan, haemodynamic evaluation, blood sampling and histology.

### Surgical and interventional procedures

All interventions were performed under general anaesthesia. The following protocol for anaesthesia was used: ketamine 22 mg/kg injected intramuscular (IM) with subsequent inhaled isoflurane 5% at induction with 2.5% isoflurane through the endotracheal tube during the procedure. For every interventional procedure, analgesia was administered using a combination of buprenorphine 0.03 mg/kg and meloxicam 0.2 mg/kg intravenously (IV). After surgical and percutaneous interventions, analgesics were continued as long as necessary. Before the thoracotomy, 40 000 IU/kg IV penicillin and 6.6 mg/kg IV gentamycin were administered.

**Figure 1:**
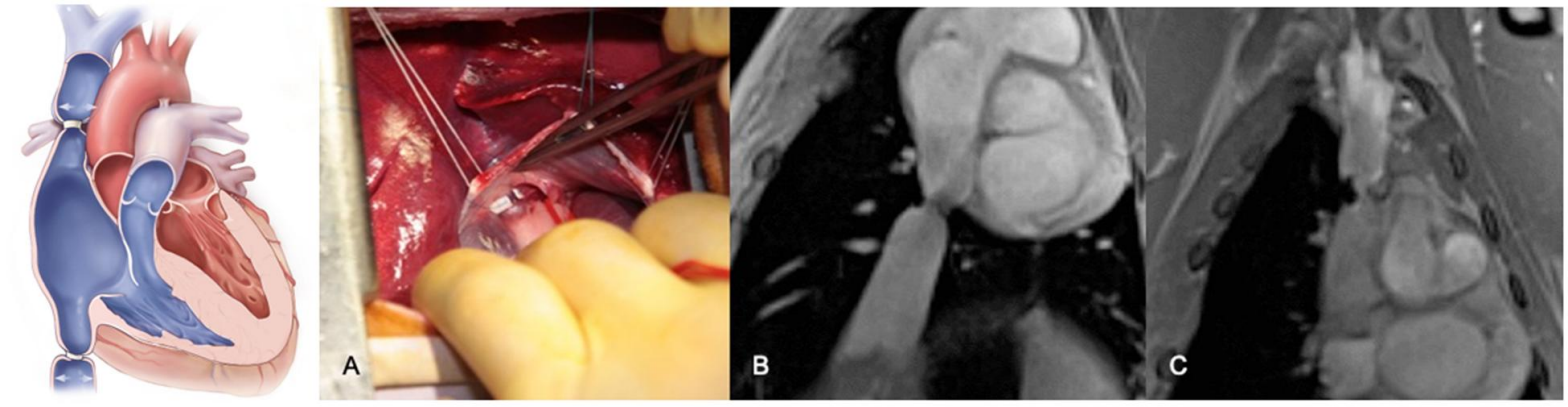
Banding of caval veins: (**A**) surgical view of the band (PTFE strip) of the SVC; (**B**) MRI showing the band of the IVC before debanding and (**C**) same for SVC.

At lamb stage 18–20 weeks old (weight of 15–25 kg), a calibrated banding of the superior and inferior caval veins (superior vena cava [SVC] and IVC) was performed. Surgery was performed using a right lateral thoracotomy in the fourth intercostal space. Both the SVC and IVC were tightly banded using a polytetrafluoroethylene (PTFE) strip at the junction of the right atrium. The length of the band was tailored to half of the measured diameter of the respective caval vein (length of the PTFE strip = π x R). The PTFE strip ends were fixed with prolene 5/0; another stitch with prolene 5/0 fixed the band on the vessel wall (Fig. 1).

Ten months after banding, haemodynamic measurements were made and percutaneous debanding was performed. Both bands were dilated to full expansion using high-pressure Atlas Gold 20 or 22 mm balloons with inflation pressure up to 18 ATM. After balloon dilation, an angiogram was performed at the SVC and the IVC levels to rule out rupture or vessel tears. All animals underwent CMR analysis in the week before the debanding procedure and within 48 h after debanding. After the last CMR analysis, animals were sacrificed, and the heart was explanted for histological analysis.

### Cardiac magnetic resonance

All animals underwent CMR analysis using a 3 T Magnetom PRISMA, Siemens Healthineers, Erlangen, Germany. All scans were performed in right decubitus position, during suspended respiration and ECG gating. Ventricular volumes, mass, stroke volume (SV) and ejection fraction (EF) were obtained. SVC and IVC flows were obtained on phase contrast images. Data were indexed for body surface area (BSA) with use of the formula of Mitchell 0.09×W^0.67^, where appropriate [10].

### Haemodynamics

Baseline measurements were carried out before debanding, and all haemodynamic measurements were repeated within 30 min after debanding. Access was gained by a 12 Fr introducer sheath (Avanti, Cordis, Baar, Switzerland) percutaneously placed in the jugular vein and a 7 Fr sheath placed by surgical cutdown into the carotid artery. A 7 Fr Swan-Ganz catheter (Edwards Lifesciences) was placed into the pulmonary artery to measure cardiac output (CO). The procedures were performed under fluoroscopy guidance.

Subsequently, pressure–volume (PV) analysis was done using CD Leycom Conduct NT (Hengelo, The Netherlands) software and conductance catheter CA-71123-PL. The CD Leycom volume calibration was done using MRI data prior to analysis. After calibration, the conductance catheter was advanced into the LV under fluoroscopic guidance. Multiple sequential PV loops during intermittent additional unloading of the ventricle were acquired at baseline condition and after debanding using a compliant 25 mm PTS sizing balloon (Numed Inc, Hopkinton NY, USA) occluding the IVC-right atrial junction to unload the right ventricle (RV).

The pressure–volume (PV) loops were analysed using MatlabR2017 (MathWorks, Inc., Natick, MA, USA). The PV loops had decent quality for analysis in 7 of the 11 animals of the intervention group. The end-diastolic PV relationship (EDPVR) and end-systolic PV relationship (ESPVR) were calculated on a beat-by-beat analysis; MRI data were used for calibration. The myocardial stiffness constant βw is derived from β*V_w_, where V_w_ is the left ventricular wall volume. The curve fit used for EDPVR is p = Ce^βV^ [11]. The volume on the EDPVR at which the pressure was 15 mmHg [V_15_ = (15/β)^1/α^] was calculated [12]. The ESPVR data were fit by linear regression analysis to arrive at the slope (E_es_, end-systolic elastance) and volume–axis intercept (V_o_): P_es_ = E_es_(V_es_ − V_o_). The volume on the ESPVR at which the pressure was 90 mmHg (V_90_ = 90/E_es_ + V_o_) was calculated [12].

### Histology

All explanted hearts were biopsied at the midventricular level of the RV and LV. The biopsies were fixed in 10% formalin followed by paraffin embedding. Of each paraffin block, sections were made at 5-μm thickness and stained with hematoxylin and eosin (HE; 3801540BBE and 3801590BBE, Leica Biosystems, Wetzlar, Germany) and Picrosirius red (PSR, without counterstain; 09400–25, Polysciences, Hirschberg an der Bergstraße, Germany and 84512.260, VWR, Radnor, PA, USA) for microscopic analysis. The samples were examined blinded by an expert pathologist. For assessment of cardiomyocyte hypertrophy, the number of myocytes transected by a 0.5 mm line was counted per microscopic field, on 10 fields per biopsy and then averaged. The number of transected myocytes correlates with the inverse of myocyte volume and thus indexes myocyte hypertrophy. Next, for quantification of the extent of fibrosis, the PSR slides were scanned (Zeiss Axio Scan.Z1, Oberkochten, Germany) and imported into QuPath software (version 0.4.3) for image analysis [13]. The ventricular lumina and any possible regions of false-positive staining were manually excluded for each biopsy. Consequently, the PSR-positive area was calculated by the software after setting a well-considered colour threshold. This PSR-positive area was divided by the total biopsy area to obtain the percentage of PSR-positivity for each animal, quantifying the extent of fibrosis.

### Statistical analysis

Statistical analysis was performed using SPSS version 26 (IBM) and tools available in Prism 10 (GraphPad). Test for normality was done using Kolmogorov–Smirnov test. Comparison among different groups was done using the non-parametric Kruskal–Wallis test with multiple comparison. Comparison of paired data was carried out using the non-parametric Mann–Whitney test. Continuous data are expressed as medians and the interquartile range (IQR). Statistical significance was accepted with a *P*-value <0.05.

## RESULTS

### Haemodynamic data

**Figure 2:**
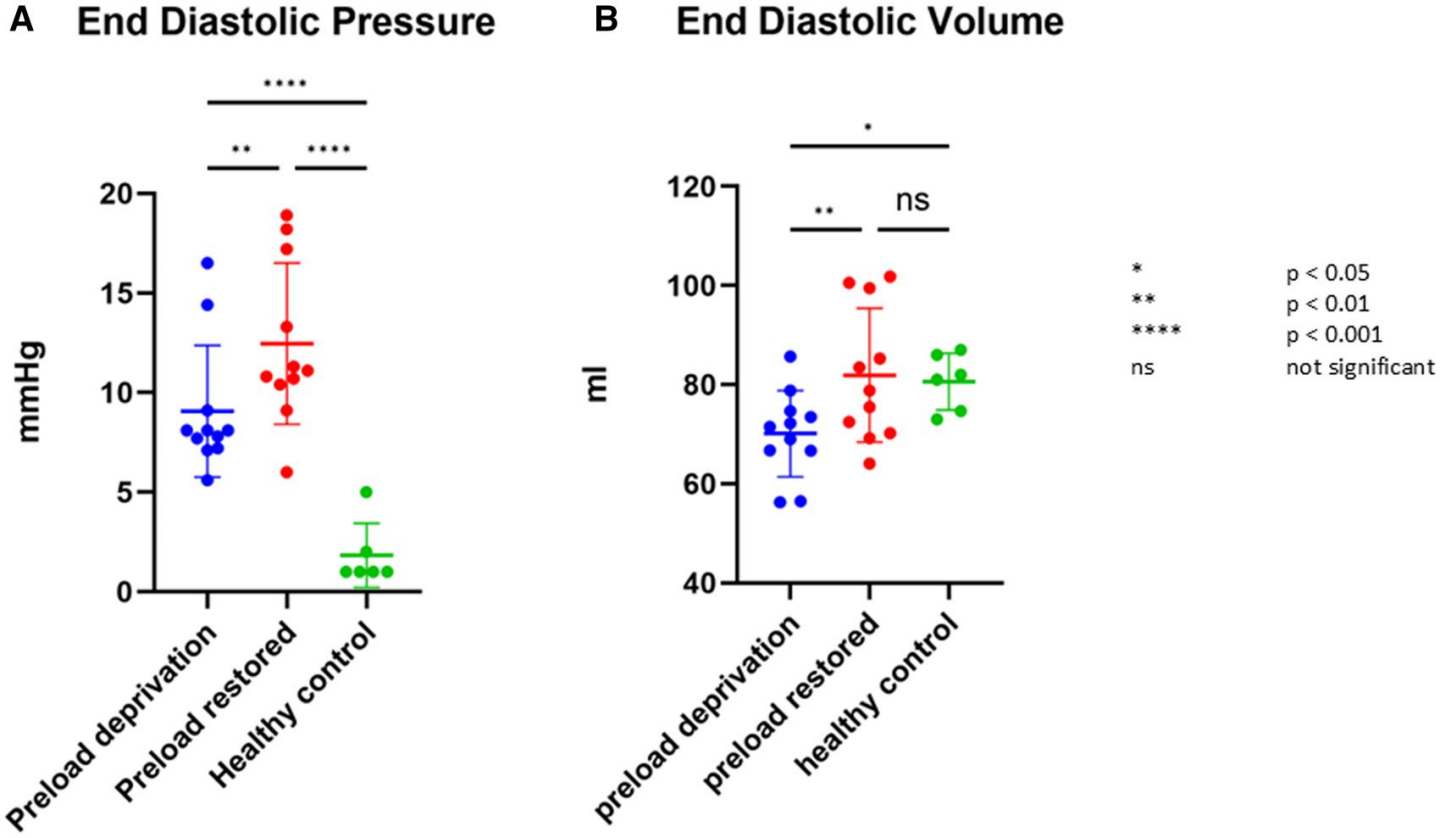
Pressure–volume comparisons. Comparison of (**A**) end-diastolic pressure (EDP) (mmHg), (**B**) end-diastolic volume (EDV) (ml) between animals in preload deprivation state and after restoring the preload compared to age- and weight-matched healthy control animals. The EDP is significantly higher in the preload-deprived animals compared to healthy animals (green) and rises significantly after restoring the preload. The EDV is significantly lower in the preload-deprived animals compared to healthy animals and increases significantly after restoring preload. After restoring preload, there is no significant difference anymore compared with healthy animals.

**Figure 3:**
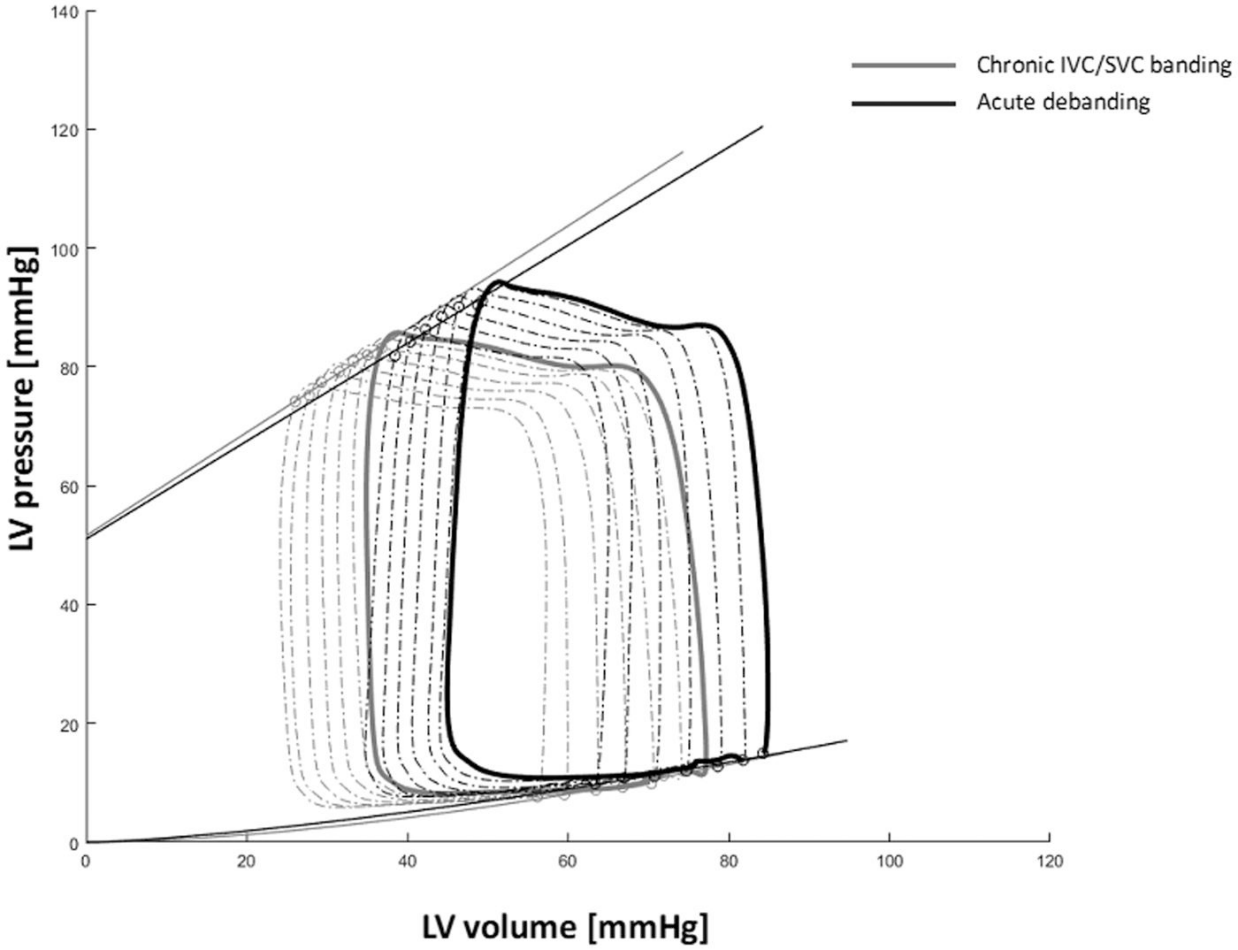
Pressure–volume curve. Pressure–volume relationship of a single animal derived from pressure–volume catheter in the left ventricle. The figure shows the PV loop (grey) with preload reduction in the animal at deprived state and the second PV loop (black) after restoring the preload. The figure shows the acute increase in volume and rise in end-diastolic pressure. The end-diastolic pressure–volume relationship (EDVPR) and the end-systolic pressure volume relations (ESPVR). The line in grey represents the deprived state, and the line in black represents the state after preload restoration.

**Figure 4:**
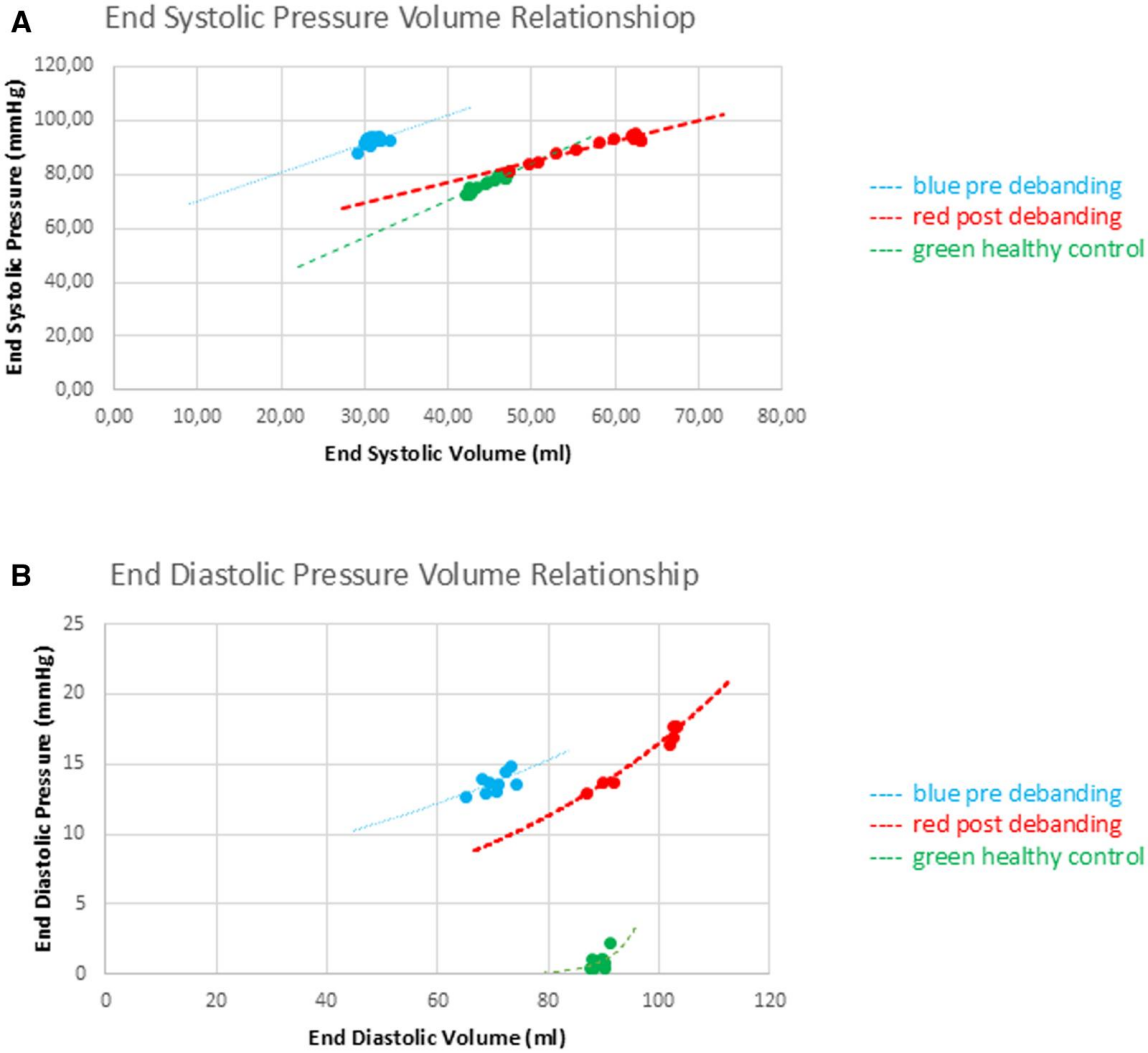
Pressure–volume relationship. End-systolic pressure–volume relationship (ESPVR) end-diastolic pressure–volume relationship (EDPVR) curves—loop of a single animal compared to a matched control. (**A**) ESPVR decreased contractility in chronic deprived ventricle compared to healthy control. No change in contractility when preload is restored. (**B**) EDPVR decreased compliance in chronic deprived ventricle compared to healthy control.

Data can be viewed in Table 1 and Figs 2–4.

**Table 1:** CMR and haemodynamic data

	Pre-debanding		Post-debanding		Healthy control		Pre post	Pre control	Post control
	Median	*IQR*	Median	*IQR*	Median	*IQR*	*P-value*	*P-value*	*P-value*
*CMR*	*n* =11		*n* =11		*n* =6				
HR (BPM)	91	80–97	101	82–112	92	87–99	0.54	0.99	0.99
EDV (ml)	71.5	66.7–74.7	78.8	70.2–99.5	81.5	74.3–86.3	0.004	0.013	0.96
ESV (ml)	34.5	27.5–37.6	45.7	35.6–48.3	42.7	35.0–50.5	0.001	0.022	0.032
SV (ml)	41	36–48	40	35–49	35	28–56	0.99	0.99	0.99
EF %	59	56–61	53	46–56	51	34–54	0.115	0.028	0.99
*Haemodynamics*	*n*=11		*n*=11		*n*=6				
CO LV (l/min)	4.1	3.2–4.8	4.1	3.4–5.0	4.0	2.9–4.2	0.99	0.80	0.81
EDP (mmHg)	8.1	7.2–9.1	11.1	10.4–17.2	1.0	1.0–2.7	0.038	0.030	0.001
ESP (mmHg)	83.4	73.1–89.0	85.7	73.6–90.9	73.3	53.5–76.5	0.99	0.105	0.194
*PV loop*	*n* = 7		*n* = 7		*n* = 6				
Ees	1.1	0.7–1.6	1.1	0.8–1.3	1.4	0.5–2.3	0.99	0.99	0.99
Beta exp	1.6	0.7–3.7	1.4	0.2–1.7	3.0	0.1–3.7	0.99	0.183	0.57
beta power	0.01	0.01–0.22	0.01	0.01–0.20	1.0	0.00–0.87	0.89	0.21	0.99
Alfa exp	0.03	0.02–0.04	0.03	0.02–0.05	0.16	0.04–0.98	0.99	0.033	0.158
Alfa power	1.6	0.9–1.9	2.3	1.8–3.2	12.4	1.3–20.1	0.77	0.195	0.99
V0	−35.6	−80.1 – 20.8	−35.4	−60.6 – 23.1	14.1	−23.1 – 58.1	0.99	0.075	0.075
V90	38.4	31.5–55.3	47.3	37.8–67.9	28.5	7.3–44.1	0.96	0.89	0.138
PRSW	35.9	24.1–54.3	44.2	29.3–48.4	44.2	4.0–65.5	0.99	0.99	0.99

The end-diastolic pressure (EDP) in the chronic volume-deprived LVs was significantly elevated in the intervention group compared to the controls; a further significant increase occurred immediately after the release of caval obstructions with acute restoration of preload: median (IQR) 8.1 (7.2–9.1) vs 1.0 (1.0–2.7) (*P* 0.030) and 11.1 (10.4–17.2) mmHg (*P* 0.038), respectively (Table 1). End-systolic pressures in the intervention group prior to debanding was 83.4 (73.1–89.0) mmHg compared to 73.3 (53.5–76.5) mmHg of the controls (*P* 0.99) and demonstrated a mild, insignificant increase after debanding to 85.7 (73.6–90.9) mmHg (*P* 0.105).

The end-diastolic volume (EDV) and end-systolic volume (ESV) of the intervention group in the deprived state were also significantly less than the healthy controls (*P* 0.013 and 0.022). The EDV of the LV increased significantly from 71.5 (66.7–74.7) to 78.8 (70.2–99.5) ml after restoration of the preload (*P* 0.004). The ESV increased significantly from 34.5 (27.5–37.6) ml to 45.7 (35.6–48.3) ml (*P* 0.001) following preload restoration. SVs and EFs of both groups were similar.

The EDPVR curves were shifted upwards and to the left compared to controls, indicative of a decreased compliance of the chronically deprived LV. The end-systolic pressure–volume relationships (ESPVR) demonstrated decreased contractility with rightward shifting of the deprived LV’s pre-release curve, which only slightly recovered after debanding compared to the control animals. However, the slopes (Ees) and preload recruitable stroke work (PRSW), although tending higher, were not statistically different compared to the controls.

Left ventricular CO measured by thermo-dilution remained unchanged at 4.1 (3.2–4.8) ml/min in the deprived state and 4.1 (3.4–5.0) ml/min after restoring preload and did not differ from the healthy controls 4.0 (2.9–4.2) ml/min. The heart rate in the intervention group increased somewhat from 91 (80–97) beats per minute (BPM) in the deprived state to 101 (82–112) BPM after restoring the preload, similar to the control group (92 [87–99] BPM).

### Histology

**Figure 5:**
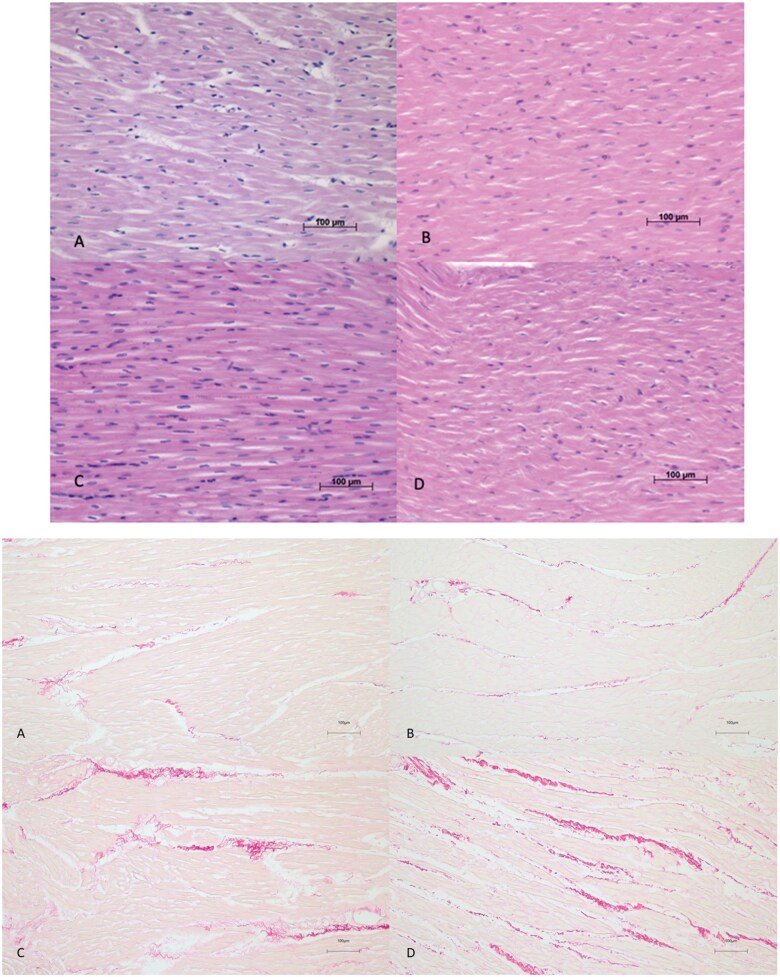
Histology. (**A**) Histology hematoxylin-eosin staining 100× magnification: (**A**) left ventricle intervention group, (**B**) left ventricle healthy control, (**C**) right ventricle intervention group, (**D**) right ventricle healthy control. (**B**) PSR staining 100× magnification: (**A**) left ventricle intervention group, (**B**) left ventricle healthy control, (**C**) right ventricle intervention group, (**D**) right ventricle healthy control.

The mean number of transected myocytes per 0.5 mm of the LV did not differ between the intervention and control sheep (16.3 [14.6–17.9] vs 18.0 [16.9–18.9] cells, *P* 0.128), but in the RV significantly less cells/mm were observed in the intervention group (17.1 [14.2–18.0] vs 20.9 [19.8–21.8] cells, *P* 0.001), which indicates RV hypertrophy in the intervention group. The LV and RV mean weights were similar in the two groups (91.0 [84.9–96.7] vs 95.3 [80.4–97.8] g). Fibrosis analysis of the LV and RV did not show a significant difference between deprived and normal ventricles, respectively (0.25%±0.10 vs 0.34%±0.20) (*P* 0.54) and (0.97%±0.64 vs 2.15%±2.59) (*P* 1.00), respectively (Fig. 5A and B).

## DISCUSSION

Knowledge of the haemodynamic changes occurring as a result of chronic volume deprivation in ventricles is limited. In this large animal ovine model, chronic left ventricular volume deprivation resulted in significantly increased EDPs, decreased ventricular volumes and higher myocardial stiffness with reduced ventricular compliance. Relief of volume deprivation resulted in the restoration of ventricular volumes, but an acute increase in EDPs was observed. Cardiac hypertrophy was observed in the RV only, and no overt fibrosis was detected despite the volumetric and haemodynamic changes.

Preload to the ventricle was limited by bi-caval banding, resulting in effective preload reduction to the systemic ventricle as indicated by the decreased EDV by ten months. During volume deprivation, we observed an increase in EDP and decreased compliance when compared to age and weight-matched animals. Acutely restoring preload to the ventricles resulted in an acute further increase in EDPs, confirming the decreased compliance. This is not surprising since, when preload is restored acutely, filling pressures rise temporarily and the capability of myocardium to increase the stretching of muscle fibers is limited in its ability to adjust in such a short space of time. This is analogous to stretching of unused skeletal muscle as previously alluded to.

Restoration of preload resulted in improved volume-pressure relationships in both diastole and systole. Systolic function was consistently maintained and comparable to the controls. Similar observations have been made in patients with severe mitral stenosis where the LV due to chronic reduced preload was shown to become less compliant and pressure–volume relationships were shifted leftwards [5, 14, 15]. The investigators observed an improvement in compliance early (hours) after restoring normal preload. After 3 months, further improvements in ventricular compliance of up to 25% were observed in these patients. The authors concluded that the diastolic dysfunction due to chronic volume restriction led to augmented stiffness of the LV, which resumed after restauration of preload. It should be noted that systolic function was not affected in these patients, similar to our results. These findings have also been described in patients following ASD closure, relief of right heart obstruction, constrictive pericarditis and following the Fontan operation [16–18]. All these conditions share a common denominator: a critical bottleneck upstream with downstream decreased flow with secondary effects. The acute flash pulmonary oedema following relief of the bottleneck in some of these conditions could be attributed to the ‘stiffness’ of the ventricle and high EDPs following chronic preload starvation. Reversal of such flash pulmonary oedema after closure of an ASD, embolectomy of long standing pulmonary hypertension, Melody valve implantation in patients with severe right heart obstruction can be treated by using judicious volume restriction, diuretics and positive end-expiratory pressures while awaiting the improvement of ventricular compliance [19].

Apart from right ventricular hypertrophy, there were no marked differences between the intervention group and the healthy controls at histological level. Therefore, the mechanism for decreased compliance is probably not fibrosis since in several models and clinical studies, stiffness normalized after restoring adequate preload [20]. An explanation might include altered viscoelastic myocardial properties (actin-myosin within the sarcomere) and pericardial constraint, as well as possible changes of chamber geometry [21–23].

What are the implications for the clinician? It is essential to understand why and how a chronically deprived ventricle can give rise to secondary haemodynamic effects as it will influence management and treatment choices. Increased awareness among cardiologists is important: in contrast to acute volume unloading, where ventricular filling pressures decrease, chronic volume deprivation will cause ventricular stiffness. Every athlete and physiotherapist knows intermittent muscle stretching is good for long-term performance. The same probably applies to the ventricle: exercise can increase the SV up to 50% with concomitant myocardial stretch. A sedentary lifestyle without exercise will result in a stiff ventricle and early accelerated ageing [23, 24]. When successful treatment is implemented by relieving the critical bottleneck and improving the preload, the clinical condition may acutely but transiently deteriorate as evidenced by flash pulmonary oedema [17, 20, 25, 26]. When assessing the degree of volume deprivation, not only the degree of volume reduction is important, but also the degree of overgrowth of the ventricle as is observed after abolishing a large shunt or regurgitant fraction that might have induced ventricular overgrowth (mitral regurgitation, aortic regurgitation, ventricular septal defect, shunts like large ductus arteriosus or aorta-pulmonary shunts in single ventricles) [1]. In Fontan patients, volume deprivation of the ventricle may reduce compliance, increase left atrial pressure, thereby reduce the transpulmonary gradient which drives the flow, resulting in further decrease flow; this negative spiral will result in accelerated late Fontan failure and death. The degree of volume deprivation can be reduced by keeping the Fontan connections wide and pulmonary impedance low, by avoiding ventricular overgrowth (as this will enhance the degree of deprivation, but adequate pulmonary flow is required to have a good catch-up growth of the pulmonary vessels), by allowing a fenestration (good for output and congestion, but bad for saturation), by judicious use of diuretics (good against congestion, but bad for output and ventricular volume deprivation) and by promoting exercise. Diastolic dysfunction can be unmasked in Fontan patients with poor functional status [27]. Miranda and co-workers elegantly demonstrated this phenomenon in adult Fontan patients during exercise haemodynamic testing [28].

However, several questions remain: What is the time frame of occurrence and resolution of ventricular stiffness? When improving preload, to avoid flash pulmonary oedema: can we predict in whom, or at least enhance awareness? What is the value of diuretics? Can we predict the need for prolonged positive-pressure ventilation?

### Limitations

We did not analyse the long-term effects after reloading the ventricle because animals were sacrificed for histology shortly after restoring the preload. Anesthesia had little or no effect on the haemodynamics since anaesthetic protocols for the intervention and control groups were similar.

## CONCLUSION

Our results confirm that, when a healthy ventricle is chronically deprived of an adequate preload, it becomes less compliant with elevated filling pressures. Acute reloading does not lead to ventricular systolic dysfunction, but in the early phase, diastolic pressure may further rise. A better understanding of this phenomenon might help to recognize, avoid and treat cases with impaired ventricular compliance.

## Data Availability

The data underlying this article will be shared on reasonable request to the corresponding author.

## ABBREVIATIONS

BPM: Beats per minute
CO: Cardiac output
EDP: End-diastolic pressure
EDPVR: End-diastolic pressure–volume relationship
EDV: End-diastolic volume
EF: Ejection fraction
ESP: End-systolic pressure
ESV: End-systolic volume
ESPVR: End-systolic pressure–volume relationship
HR: Heart rate
IM: Intra muscular
IV: Intravenous
IVC: Inferior vena cava
LV: Left ventricle
PSRW: Preload recruitable stroke work
PV: Pressure–volume
RV: Right ventricle
SV: Stroke volume
SVC: Superior vena cava
